# Rodent Models for the Analysis of Tissue Clock Function in Metabolic Rhythms Research

**DOI:** 10.3389/fendo.2017.00027

**Published:** 2017-02-13

**Authors:** Anthony H. Tsang, Mariana Astiz, Brinja Leinweber, Henrik Oster

**Affiliations:** ^1^Chronophysiology Group, Medical Department I, University of Lübeck, Lübeck, Germany; ^2^Department of Clinical Biochemistry, Institute of Metabolic Science, University of Cambridge, Cambridge, UK

**Keywords:** clock genes, metabolism, gene targeting, *Bmal1*, conditional knockout, CRE-*loxP* system

## Abstract

The circadian timing system consists on a distributed network of cellular clocks that together coordinate 24-h rhythms of physiology and behavior. Clock function and metabolism are tightly coupled, from the cellular to the organismal level. Genetic and non-genetic approaches in rodents have been employed to study circadian clock function in the living organism. Due to the ubiquitous expression of clock genes and the intricate interaction between the circadian system and energy metabolism, genetic approaches targeting specific tissue clocks have been used to assess their contribution in systemic metabolic processes. However, special requirements regarding specificity and efficiency have to be met to allow for valid conclusions from such studies. In this review, we provide a brief summary of different approaches developed for dissecting tissue clock function in the metabolic context in rodents, compare their strengths and weaknesses, and suggest new strategies in assessing tissue clock output and the consequences of circadian clock disruption *in vivo*.

## Introduction

A network of cellular clocks adapts physiology and behavior to a 24-h rhythmic environment. Epidemiological evidence suggests a strong association between circadian rhythm disruption—e.g., in shift workers—and metabolic disorders (1). The development of genetic rodent models has been essential in deciphering the mechanisms linking clock dysfunction and the pathogenesis of metabolic diseases, highlighting the therapeutic potential of circadian rhythm manipulation in this context. Here, we provide a brief overview of the metabolic consequences of circadian disruption derived from rodent model studies and discuss potential and pitfalls of emerging genetic techniques in studying clock-metabolism crosstalk.

## Non-Genetic Circadian Disruption Models

Early approaches describing the interplay between circadian clock function and metabolism were based on models of non-genetic circadian disruption (Table 1). Lesion experiments identified the suprachiasmatic nucleus (SCN) as the central circadian pacemaker (2, 3). In rats, SCN lesions abolish plasma rhythms of glucose, insulin (4), and leptin (5). In terms of general energy homeostasis, there are differences between studies that range from slight weight reductions (6) to no effect (5) and marked obesity (7). In the latter study on mice, increased weight is accompanied by hepatic insulin resistance. In sum, these studies illustrate that the SCN regulates metabolic hormones and tissue physiology either directly or *via* its control on food intake rhythms.

**Table 1:** **Advantages and disadvantages of different clock targeting approaches in rodents**.

Paradigm	Advantages	Disadvantages
Non-genetic clock disruption	No gene targeting necessary No developmental effects At least partly reversible	Non-specific (except for lesioning) and non-targeted (very broad intervention)

Classical (global) gene targeting	High recombination efficiency	No spatio-temporal control Possible developmental effects Irreversible and non-tunable

Conventional CRE-*loxP* gene targeting	Relatively high recombination efficiency	Relative tissue specificity Possible developmental effects Irreversible and non-tunable

Inducible CRE-*loxP* gene targeting	Exclude developmental effects	Relative tissue specificity Irreversible and non-tunable Reduced recombination efficiency

Chemogenetics	Reversible, tunable, and good temporal control (depending on the pharmacokinetics of the drug used)	Drug administration can interfere with the experiment Poor tissue specificity (unless combined with the use of CRE-driver mice and/or viral transgene delivery)

Optogenetics	Very good tissue specificity with implantation of light sources (when combined with the use of CRE-driver mice and/or viral transgene delivery) Reversible, tunable, and excellent temporal resolution	Phototoxicity for extended activation Technically demanding and only few (mainly CNS) tissues are applicable in mammals

In an alternative paradigm, exposure to altered light regimens leads to disruption of behavioral circadian rhythms and energy metabolism. Mice exposed to bright day/dim night illumination lose their feeding rhythm along with reduced glucose tolerance and increased body weight (8). Constant light (LL)-exposed mice develop a higher body weight together with a profound loss in insulin sensitivity (7). In rats, LL exposure disrupts pancreatic beta cell clocks, correlating with reduced glucose-stimulated insulin secretion (9). In type-2 diabetes-prone rats, LL accelerates disease development due to a 50% reduction in beta cell mass (10). Most of these studies interpret their findings as a consequence of the disruptive effects of abnormal light exposure on SCN function. However, light can alter various centrally controlled functions such as mood and appetite independent of a clock effect (11, 12). Moreover, even though irregular light regimens are common for many animals and humans, there are only very few occasions where continuous light exposure above a certain threshold level may occur, making LL a highly artificial paradigm.

Several studies have outlined shift-working as a risk factor for the development of metabolic diseases (13), and various animal models have been developed to mimic this condition. Mice exposed to repeated 6-h advances of the light/dark (LD) cycle gain significantly more body weight than stable-LD controls (14). Interestingly, when mice are exposed to paradigms mimicking even more rapidly rotating weekly shift-work patterns, there is no or only a moderate effect on body weight (15). Sleep-restricting mice during their normal rest phase for 2 weeks leads to significant alterations in liver clock gene expression rhythms associated impaired pyruvate-stimulated gluconeogenesis, but no significant alterations in body weight (16). A similar approach in rats further yielded body weight increases and impaired glucose tolerance (17).

The mixed metabolic outcomes in the mentioned shift-work studies are likely directly related to the ability of the chosen paradigms to provoke food intake during the normal rest phase. Rest phase-restricted food access in mice promotes weight gain without increased energy intake (18). In line with that, while both the rapid-shift (15) and our sleep-restriction study in mice (16) showed no change in food intake rhythms, the 6-h shift model (14) and the rat sleep-restriction study (17) reported misaligned food intake. Considering the reciprocal interaction between energy intake and clock function, it has been speculated that epigenetic programming may be an important mechanism in the circadian regulation of energy metabolism—even across generations [reviewed in Ref. (19, 20)].

## Metabolic Alterations in Conventional Clock Gene Mutant Mice

The development of clock gene mutant mouse models has provided a substantial tool to understand clock-metabolism interaction at the molecular level. Following the identification of *circadian locomotor output cycles kaput* (*Clock*) (21), further mammalian *clock genes* have been cloned and functionally characterized in corresponding mouse mutants (22–25). So far, brain and muscle ARNT-like 1 (BMAL1) has been identified as the only essential component of the molecular clockwork in mammals. Deletion of *Bmal1* in mice abolishes behavioral circadian rhythms in constant environmental conditions (25). Gene expression profiling experiments show that the rate-limiting steps of various metabolic pathways are subject to circadian regulation (26). *Bmal1* knockout (KO) mice show impaired glucose metabolism and insulin hypersensitivity (27, 28). At young age, they also gain weight more rapidly than wild-type littermates (28). Recently, an inducible global *Bmal1* KO mouse model has been developed. Unexpectedly, adult-onset *Bmal1* KOs do not suffer from many metabolic abnormalities described in the standard *Bmal1* KOs (29).

Mice expressing a dominant-negative CLOCK variant (CLOCK-Δ19) on a C57BL/6J genetic background display altered 24-h feeding patterns (30). *Clock-Δ19* mice are hyperphagic and show reduced energy expenditure. They also develop hyperglycemia, hyperlipidemia, hyperleptinemia, hypoinsulinemia, and increase in body weight and visceral adiposity under different diet conditions. These phenotypes may be partly explained by a reduced lipolytic capacity of white adipose tissue (31). By contrast, on an ICR genetic background, the same *Clock-Δ19* mutation leads to a reduction in body weight and impaired dietary lipid absorption, suggesting that the genetic background influences clock-metabolism interaction (32).

Inconsistent findings were also reported for the metabolic consequences of mutations in another clock gene, *Period (Per) 2*. *Per2^Brdm1^* mutant mice (24) show hyperphagy, diet-induced obesity (33), hyperinsulinemia with altered insulin sensitivity, hypoglycemia, and low fasting hepatic glycogen content (34). By contrast, *Per2^ldc^* mutants (35) show reduced adiposity, increased fatty acid oxidation, and hypotriglyceridemia (36), but are normoglycemic with improved clearance after glucose challenge (37). Of note, while genetic background may also play a role here, residual protein-coding transcripts have been detected in both mutants that may yield biologically active peptides (35, 38). Male, but not female, *Per1/2/3* triple mutants become obese under an HFD (39). A *Per3* KO alone has even stronger effects on male diet-induced weight gain, suggesting a genetic interaction of different *Per* genes in metabolic regulation (39).

Liver transcriptomic analyses from mice carrying *Rev-Erbα* loss- or gain-of-function alleles have identified it as a circadian regulator of cholesterol/lipid and bile acid homeostasis (40). In line with this, mice with adult-onset global loss of *Rev-Erbα/β* show deregulated glucose and lipid metabolism (41). Moreover, administration of REV-ERB agonists in mice induces body weight loss and decreased lipogenesis in liver and white adipose tissue, increased lipid and glucose oxidation in skeletal muscle, and elevated energy expenditure (42).

Together, these studies provide evidence that the circadian clock plays a fundamental role in energy homeostasis and that different clock components have specific functions in this context. However, because the mice used in these studies carry clock gene mutations in all tissues including the SCN, much like the non-genetic models discussed above, metabolic phenotypes may be confounded by systemic abnormalities such as altered sleep patterns, activity and feeding behaviors, or counteractive consequences of clock disruption in different tissues.

## Tissue Clock Function in Metabolic Regulation

Several techniques have been developed to study the physiology of tissue circadian clocks (Table 1). *In vitro* experiments allow the study of tissue rhythms in the absence of external influences (43). However, such approaches have only limited potential for predicting the impact of tissue clock disruption on complex physiological processes such as energy metabolism. Transplantations have been used to study tissue clock function (44, 45). However, such techniques are highly invasive and only applicable for few tissues. Instead, conditional CRE-*loxP*-based gene targeting has been widely used for tissue-specific deletion of clock function *in vivo* (in most cases by targeting *Bmal1*) (46, 47). Here, we highlight some studies which have provided important insights into the contribution of different tissue clocks to metabolism.

Hepatocyte-specific deletion of *Bmal1* (L-Bmal1 KO) improves glucose tolerance—the opposite effect of a global *Bmal1* KO—which was attributed to reduced hepatic glucose export during the fasting phase *via* glucose transporter 2 (28). By contrast, mice with pancreatic beta cell-specific *Bmal1* deletion (P-Bmal1 KOs) show hyperglycemia and impaired glucose tolerance (48) due to decreased insulin exocytosis (49). Further, while mice with muscle-specific deletion of *Bmal1* (M-Bmal1 KO) show no significant change in systemic glucose regulation, impaired myocyte glucose uptake, and metabolism have been reported (50). Finally—and similar to global *Bmal1* KOs and *Clock-Δ19* mutants—mice with adipocyte-specific *Bmal1* deletion (A-*Bmal1* KOs) become obese (*ca*. 20% body weight) under HFD but not normal chow conditions. This phenotype correlates with misaligned food intake and blunted secretion rhythms of appetite-regulating polyunsaturated fatty acids from adipocytes (51).

Clock gene rhythms have been reported in central energy regulatory circuits (52). So far, few studies have addressed the biological function of specific brain clocks. With regard to metabolism, deletion of *Bmal1* in steroidogenic neurons of the ventromedial hypothalamus reduces sympathetic activation of thermogenesis in brown adipose tissue (53).

These few examples make clear that tissue-specific dissection of clock gene function has provided a much more detailed picture on how circadian clocks affect energy metabolism *in vivo*. Nevertheless, conclusions drawn from these experiments are often confounded by intrinsic drawbacks of the classical CRE*-loxP* system which novel genetic tools may help to overcome.

## Limitations of Classical Genetic Models in Chronobiology Research

### Poor Specificity of the CRE Driver

In CRE*-loxP*-based gene targeting, the tissue-specific mutation is determined by the transcription dynamics of the CRE-expressing promoter. While this may not be such an issue for processes confined to specific tissues, for the ubiquitously active circadian system, this poses an important limitation: many allegedly *tissue-specific* promoters show varying amounts of off-target activity. For example, for two of the studies mentioned above, CRE drivers with off-target activities have been employed, namely *Fabp4-Cre* for A-*Bmal1* and *Pdx1-Cre* for P-*Bmal1* KOs. Critically, their expression in several metabolism-regulating circuits in the brain has been documented (54). Though the authors of these studies used different approaches to address this problem (such as supplementing *in vitro*/*ex vivo* data or repeating key experiments with another CRE driver), certain conclusions drawn from the *in vivo* metabolic experiments may still be ambiguous. Similarly, in the hypothalamic steroidogenic neuron *Bmal1* KO study, the *Sf1-Cre* line used may very likely affect steroidogenic cells in other tissues (53).

### Developmental Compensation

Some CRE drivers are developmentally active and clock genes are known to be involved in early developmental processes (55). Therefore, some phenotypes observed in adult mice might reflect changes in developmental programs while others may be masked by compensatory responses. A strategy circumventing this issue is to employ an inducible version of CRE (T2-CRE), which is activated only when animals receive tamoxifen (56). This approach has been used to globally delete *Bmal1* and *Rev-erbα/β* (29, 41). In the M-*Bmal1* KO study discussed above, a muscle strength phenotype was only observed in mutants with conventional, but not inducible CRE deletion (50).

### Incomplete Recombination

Due to variations in expression levels or epigenetic effects, CRE-mediated recombination frequently does not occur in all cells of the same tissue. This issue is further exaggerated with the T2-CRE system. Thus, the lack of certain phenotypes in tissue-specific mutants may stem from non-recombined cells sufficient to maintain tissue function. In the circadian clock context, this issue becomes critical in tissues of strong intercellular coupling such as the SCN. Our own approach to target SCN pacemaker function using a Syt10-Cre-driver line revealed that only with the highest CRE dosage (*Syt10^Cre/Cre^*) and on a *Bmal1^flox/del^* background, recombination was sufficient to ablate behavioral rhythmicity (57).

### The Non-Circadian Role of Individual Clock Genes

Many clock gene mutant studies fail to discern whether a phenotype is caused by altered clock rhythmicity or by loss of a specific clock component. For example, in the L-*Bmal1* KO study, the mutation not only abolishes the transcription rhythm of *Glut2*, but also dramatically downregulates its overall expression (28). The multifaceted phenotype of *Bmal1* KO mice suggests that this gene has important functions outside the circadian timekeeping system (58). Along this line, the monopolized use of *Bmal1* targeting for tissue-specific clock deletion further exacerbates this issue since some of the reported phenotypes may be caused by a loss of *Bmal1* rather than of the clockwork.

## Emerging Genetic Techniques for Chronobiology Research

Some of the emerging novel genetic techniques may help to overcome the issues discussed above. Below we will highlight some methodologies that may help improving our understanding of the role of tissue clocks in complex physiological contexts.

### Improving the Spatio-Temporal Resolution of Clock Gene Manipulation

Though pharmacologically inducible CRE systems bypass developmental effects, this strategy often compromises the recombination efficiency and complicates experimental designs (56). Moreover, the manipulation is irreversible, rendering it unsuitable for certain biological questions. As an alternative approach, chemogenetic manipulations such as tetracycline-based (i.e., Tet-ON/OFF) systems achieve anatomical specificity by expressing effectors under tissue-specific promoters, the activity of which depends on the presence/absence of an otherwise inert chemical (59, 60). These systems are reversible and have a very flexible space-time window for manipulations. For example, two complementary studies have established mouse lines expressing *Clock-Δ19* and *Rev-erbα* as clock disruptors in brain and liver, respectively, in a tetracycline-dependent manner, to elucidate the relative contribution of central and peripheral clocks to physiological rhythms (61, 62). This revealed that circadian expression rhythms of most rhythmic transcripts in the liver depend on local oscillators whereas 10% of the rhythmic transcripts (including *Per2*) are sustained by systemic signals.

Optogenetic techniques have recently been introduced in chronobiology research. Photic stimulation on channelrhodopsin-2-expressing SCN neurons results in phase-resetting of SCN firing and behavioral rhythms (63). Other optogenetic systems allow for photo-switchable control of specific cellular and molecular processes (64). Of note, one strategy employs modified photo-sensitive plant cryptochrome 2 clock proteins from *Arabidopsis* (64), which would allow to directly affect molecular clock function by light. In addition to high spatial control (by directing light exposure), optogenetics may benefit chronobiology research because of their supreme temporal resolution, e.g., in phase-resetting experiments.

While standard viral transgene delivery approaches using short tissue-specific promoters often fall short to confer sufficient specificity (65), this is circumvented by combining virus injections with CRE-driver mouse lines targeting transgene expression to specific cells and tissues (66). Viral approaches further benefit from high spatio-temporal control through injection time/sites and viral capsid serotypes. They, however, often suffer from somewhat reduced penetrance and technical variability (67). Viruses can be used in conjugation with chemogenetic or optogenetic manipulations to interrogate the role of circadian clock in metabolic regulation and other physiological systems with unprecedented spatio-temporal resolution.

### Perspectives for Developing Animal Models That Can Dissociate Circadian and Non-Circadian Functions of Clock Genes

Clock gene knockdown/KO experiments often cannot distinguish between circadian (i.e., timing related) and non-circadian effects of a given mutation. This may to some extent be addressed by comparing phenotypes between mutants of different clock genes. However, because of the interactive nature of clock genes this may often not yield further insights. As an alternative approach, the direct modulation of circadian period length without abolishing clock function itself in a tissue-specific manner is achievable *via* manipulating period-determining genes such as casein kinase I ϵ (44). To some extent, this and other similar approaches still inevitably change clock protein levels and, thus, pleiotropic clock gene output.

An ideal animal model would be one with altered phasing of clock gene expression rhythms but unaffected overall clock protein abundance. Generating such model has become possible with recent developments in genome editing such as CRISPR and TALEN (68, 69). CRISPR techniques have been used to “cure” retinal degeneration models in rats (70, 71). Together with the expanding knowledge of the role of the cis-regulatory elements of clock genes in determining the phase of expression rhythms, such as the identification of the phase-determining intronic enhancer of *Cry1* (72), *in vivo* genome editing may allow to selectively manipulate clock gene phasing.

## Conclusion

**Figure 1:**
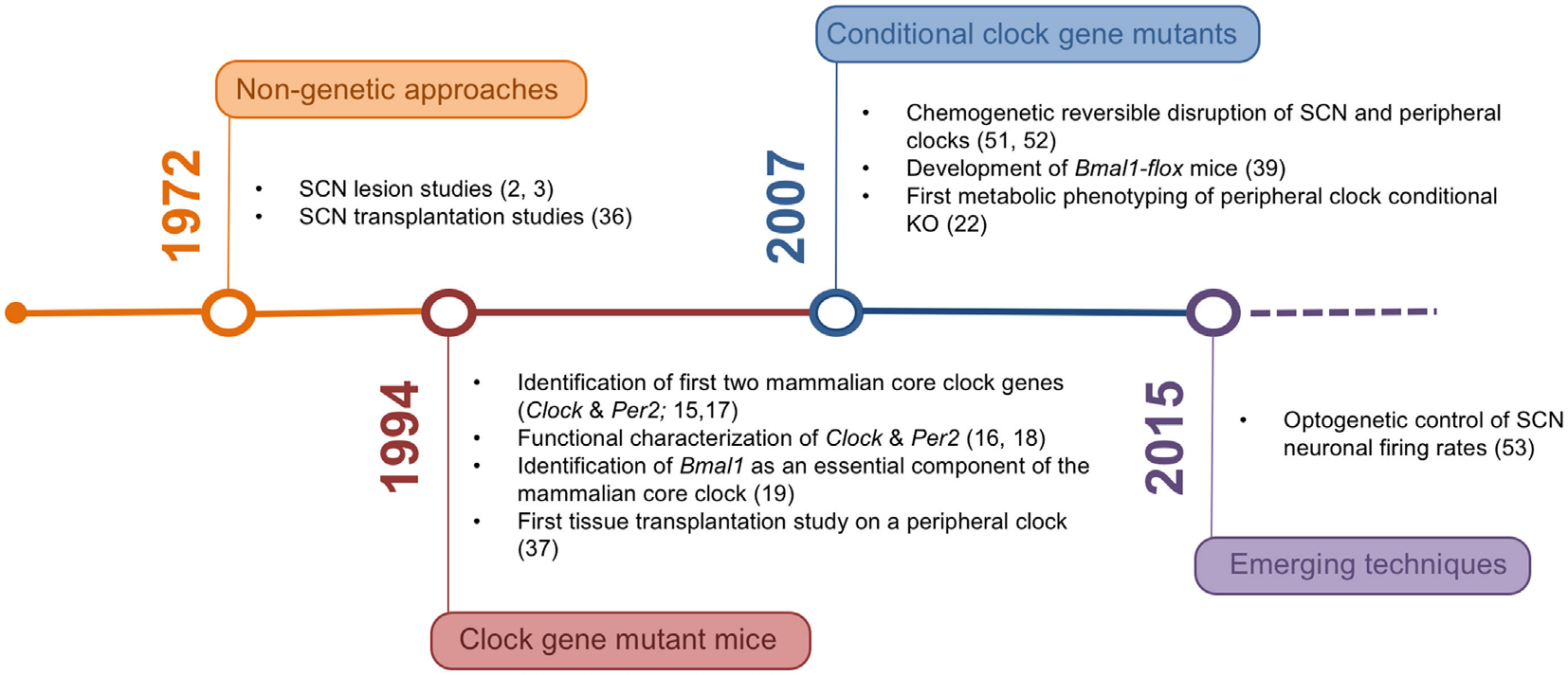
**Timeline of the development of experimental rodent models and corresponding milestone papers in circadian tissue clock research**.

In the last decades, the tools for studying the mechanisms underlying organismal circadian timekeeping and its role in metabolic regulation have been constantly refined (Figure 1). The network structure of the circadian system represents an important challenge in this context, posing high demands on both tempo-spatial control and recombination efficiency in genetic experiments. Novel genetic approaches may help to overcome these issues and provide a clearer picture of the complex interaction of different tissue clocks in the regulation of energy metabolism.

## Author Contributions

AT, MA, BL, and HO discussed the concept, complied the literature, and wrote the paper.

## Conflict of Interest Statement

The authors declare that the research was conducted in the absence of any commercial or financial relationships that could be construed as a potential conflict of interest.
